# The Pune Rural Intervention in Young Adolescents (PRIYA) study: design and methods of a randomised controlled trial

**DOI:** 10.1186/s40795-017-0143-5

**Published:** 2017-05-08

**Authors:** Kalyanaraman Kumaran, Pallavi Yajnik, Himangi Lubree, Charudatta Joglekar, Dattatray Bhat, Prachi Katre, Suyog Joshi, Rasika Ladkat, Caroline Fall, Chittaranjan Yajnik

**Affiliations:** 1 Diabetes Unit, KEM Hospital Research Centre, Rasta Peth, Pune 411011 India; 2 MRC Lifecourse Epidemiology Unit, University of Southampton, Southampton, SO16 6YD UK

**Keywords:** Cord Blood, Data Safety Monitoring Board, Multiple Micronutrient, Indian Baby, High Cord Blood

## Abstract

**Background:**

The Pune Maternal Nutrition Study (PMNS) was established to prospectively study the relationship of maternal nutrition to fetal growth and later cardiometabolic risk in the offspring. High homocysteine and low vitamin B12 levels in pregnancy predicted lower birthweight and higher insulin resistance at 6 years in the offspring. B12 deficiency was widespread in this population, due to low dietary intake. We therefore commenced a community-based intervention study with the underlying hypothesis that vitamin B12 supplementation of adolescent members of the PMNS cohort will improve birth weight, B12 status, and reduce future diabetes risk, in their offspring.

**Methods:**

The individually randomised controlled trial commenced in September 2012, with boys and girls randomized into 3 groups, to receive daily for at least 3 years or until the birth of their first child: 1) vitamin B12 2 μg; or 2) vitamin B12 2 μg plus multiple micronutrients (MMN) plus 20 g of milk powder or 3) placebo. Iron and folic acid is given to all participants. Compliance is assessed by monthly supplement counts. Adverse events are recorded using a standardised questionnaire. The primary outcome is cord blood B12 concentration; based on 180–200 pregnancies in the girls, the study has ~80% power to detect a 0.5 SD change in newborn B12, in the B12 supplementation groups compared with controls, at the 5% significance level. Primary analysis will be by intention to treat.

**Discussion:**

Our study tests a primordial prevention strategy through an intergenerational intervention started pre-conceptionally in both boys and girls using physiological doses of micronutrients to improve immediate pregnancy-related and long-term cardio metabolic outcomes. The results will have significant public health implications in a setting with widespread B12 deficiency but relative folate sufficiency. The randomised controlled trial design allows us to be confident that our findings will be causally relevant.

**Trial registration:**

ISRCTN 32921044, applied on 14/09/2012. CTRI 2012/12/003212, registered on 02/12/2012. Retrospectively registered.

## Background

Current preventive strategies to reduce the burden of type 2 diabetes mellitus (T2DM) focus mainly on improving the lifestyle of middle-aged individuals with pre-existing disease or risk factors. However, these measures can at best be termed secondary prevention and do not address the impact of the disease on future generations. So far, genetic research has also failed to provide any major breakthrough towards primary prevention.

David Barker’s ‘fetal programming’ hypothesis [1] suggests that alterations in the nutritional supply during critical stages of intrauterine growth permanently alter the structure and function of the fetal organs and offers a novel primary prevention strategy. This idea was based on findings, initially in the UK, that the prevalence of coronary heart disease, T2DM and impaired glucose tolerance was higher in adults who had lower birth weights [2]. Subsequently, these findings were replicated in different parts of the world, including India [3–7]. This suggested a possible role for fetal under-nutrition in the aetiology of these conditions. Postnatal growth is also associated with risk of T2DM; individuals with the highest risk are those who had a low birth weight but developed a high body mass index (BMI) as children or adults [8]. The fetal programming hypothesis was expanded to include postnatal factors and evolved into the ‘developmental origins of health and disease’ hypothesis (DOHaD).

The DOHaD hypothesis is supported by animal studies showing that obesity, hypertension and diabetes can be programmed by under- or over-nourishing the fetus by changing the pregnant mother’s diet [9, 10]. In contrast, evidence for DOHaD in humans is largely limited to observational studies using either extreme nutritional situations (eg. famine) or low birth weight as exposures, with little specific information on maternal nutrition. Follow up of children whose mothers participated in nutritional supplementation trials are limited. In India, offspring of mothers who were supplemented with energy and protein during pregnancy had lower insulin resistance and arterial stiffness in adolescence [11]. In Nepal, offspring of mothers who received multiple micronutrients (MMN) had lower blood pressure [12]. However, there were no effects on the body composition or blood pressure of offspring of women who received energy and protein supplementation in the Gambia [13].

Recent research suggests that programming effects may act through ‘epigenetic’ mechanisms that alter expression of genes without altering the DNA base sequence (i.e., they change the phenotype without changing the genotype). Epigenetic mechanisms may include changes in the methylation of DNA, modification (acetylation) of histones, and through miRNAs [14]. Animal studies have shown that nutritional interventions in pregnant mothers induce permanent changes in DNA methylation, gene expression, and later phenotype of the offspring [15, 16]. Epigenetic studies in humans are limited but maternal nutrition has been shown to be associated with gene-specific differences in DNA methylation [17, 18], and DNA methylation in cord blood has been related to later adiposity [19, 20]. Nutrients that are required for the activity of the 1-Carbon (1-C) cycle, such as folate and vitamin B12, may be particularly relevant in relation to epigenetics, because the 1-C cycle generates methyl groups used in DNA methylation reactions. Research in The Gambia, where there is marked seasonal variation in maternal nutritional status, has shown that maternal levels of several 1-C-related nutrients around the time of conception are related to DNA methylation signatures in the children [21, 22].

The DOHaD concept offers a paradigm shift in thinking about the aetiology, and therefore prevention, of diabetes and cardiovascular disease, and may be particularly applicable to the Indian situation characterised by a high burden of maternal malnutrition and low birth weight [23]. India is also one of the diabetes capitals of the world, with an estimated ~72 million people with T2DM in 2013, expected to rise to ~123 million by 2035 [24].

In 1993, we set up the Pune Maternal Nutrition Study (PMNS) in a rural community in western India to prospectively investigate the relationship between maternal nutrition and long-term cardiometabolic outcomes in the children. This study demonstrated a link between maternal micronutrient intake and offspring size; mothers who ate green leafy vegetables, fruit and milk more frequently, and who had higher red cell folate levels gave birth to heavier babies [25]. Maternal energy and protein intakes were unrelated to newborn size. When the Indian babies were compared to babies born in the UK, the Indian babies were 700 g lighter but had comparable subscapular skinfold thickness. Further studies using whole body MRI in the new-born confirmed that Indian babies have more abdominal fat at birth than English babies [26], and higher cord blood insulin and leptin concentrations [27]. This demonstrated that the ‘thin-fat’ Indian phenotype (a small body size with disproportionately high adiposity) [28] was present at birth and was not a consequence of post-natal life. Lower maternal B12 in pregnancy was also associated with higher maternal total homocysteine (tHcy) which predicted lower birth weight [29]. Mendelian Randomisation techniques analysing MTHFR genotypes predicting tHcy (C677T) suggested a possible causal link for the inverse association between tHcy concentrations and birth weight [30].

Higher folate concentrations in the mothers during pregnancy predicted greater adiposity in their children at 6 years of age [31]. Lower B12 in the pregnant mothers was associated with increased insulin resistance in their children; the highest insulin resistance was in children born to mothers with low B12 and high folate during pregnancy [31]. These findings were replicated in a study in Nepal [32]. About 70% of PMNS mothers had low B12 status during pregnancy (plasma concentration <150 pmol/l) and 31% had a B12 concentration of <100 pmol/l. In contrast, less than 1% had low erythrocyte folate status (<283 nmol/l) while 30% had high homocysteine (tHcy) (>10 μmol/l) an indicator of deficiency of B12 or folate or imbalance of the two [29].

Subsequent work has shown that B12 deficiency in this community is due to low intakes, rather than to malabsorption [33]. A pilot intervention in the same community showed that B12 status can be improved, and tHcy reduced, using physiological doses (2 μg/day) of B12 for one year [34]. We therefore commenced a randomised controlled trial within the cohort as the next logical step to investigate whether improving maternal B12 status improves fetal B12 status and growth, and reduces long term cardio metabolic risk in the next generation.

Previous intervention studies of maternal supplementation started in mid-pregnancy and would have missed the processes that occur around conception and early-gestation such as, gametogenesis, fertilisation, fetal epigenetic reprogramming, embryogenesis, placentation, and organogenesis. We therefore decided to start the intervention pre-conceptionally to influence these processes.

## Objectives

The main objectives are to determine whether pre-conceptional B12 supplementation of adolescents/young adults:

- improves the B12 status of their new-borns
- improves birth weight, neonatal body composition (increased lean mass and reduced adiposity), insulin sensitivity and cognitive function in the children
- alters the methylome, transcriptome, and metabolome in the cord blood of babies born in the trial.

## Methods

### Study population

**Fig. 1:**
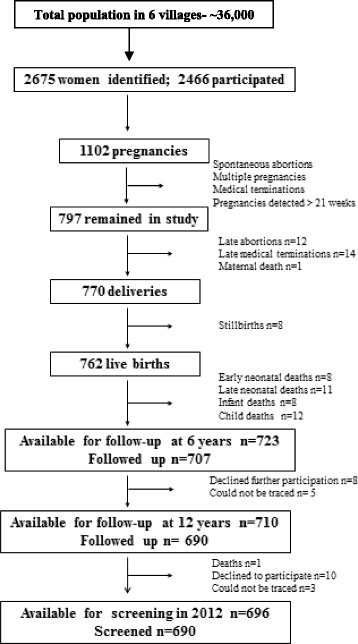
Flow of participants in the Pune Maternal Nutrition Study leading to the current trial

**Fig. 2:**
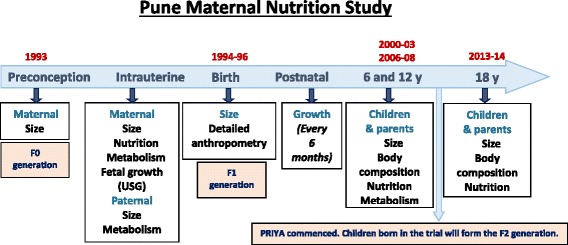
Measurements and data available at each stage in the Pune Maternal Nutrition Study. A biobank of stored plasma, serum, DNA, urine and stool samples are available from parents (F0) and offspring (F1) at various time points. Cord blood and placenta samples from the children born in the trial (F2) are collected for genetics and epigenetics, immunophenotyping, and microbiota

The Pune Maternal Nutrition Study (PMNS) was set up in 1993 [25]. At that time, we identified 2,675 married non-pregnant women in 6 villages near Pune for possible enrolment in the study, of whom 2,466 consented to take part. One thousand one hundred and two pregnancies were identified and of these, women with a singleton pregnancy of less than 21 weeks gestation (*n* = 797) entered the study and were assessed for fetal growth, diet and micronutrient status (Fig. 1). The study started in September 1993, the first pregnancy was enrolled in June 1994, and the last delivery occurred in April 1996. Of the 797 mothers enrolled in the study, 12 had spontaneous abortions, 14 terminated their pregnancy, 8 babies were stillborn and 1 mother died during pregnancy. Thus 762 mothers delivered in the study of whom 9 had babies with major congenital anomalies. The remaining children had detailed anthropometry at birth (weight, length, head, mid-upper-arm and abdominal circumferences and skinfolds). Anthropometric data were collected every 6 months, and more detailed measurements of body composition and cardio metabolic risk factors were performed at 6 years, 12 years and 18 years of age (Fig. 2).

Based on the associations between maternal nutrition and cardio metabolic risk in the offspring described above, we decided to carry out the micronutrient trial in these boys and girls because they had reached adolescence and we had data across their lifecourse. Recent evidence suggests that the nutritional status of fathers influences epigenetic processes in their offspring [35] and we therefore included boys as well as girls in the trial.

In this rural community, girls were expected to marry and start families quite young. Local statistics showed that, although the legal age of marriage for girls is 18 years, the average age of marriage was 17, and average age at first delivery 19. For boys these numbers were 21 and 24 respectively. We anticipated that approximately 180–200 girls (and less than half that number of boys) would marry and have children within four years of starting the trial.

### Study design

**Fig. 3:**
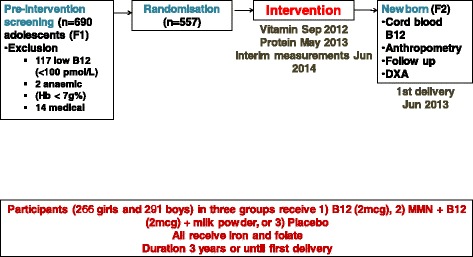
The design of the Pune Rural Intervention in Young Adults study

The design is a randomised placebo controlled trial with three arms (Fig. 3). Participants were randomised individually into one of three groups to receive either: i) 2 μg B12, ii) 2 μg B12 plus multiple micronutrients (MMN) plus 20 g milk powder (equivalent to 5 g milk protein), or iii) placebo daily.

The MMN plus protein group is an important pragmatic strategy. Studies in rural India have shown that micronutrient deficiencies are usually multiple and that protein intakes are below recommended levels [25, 36]. We wanted to ensure that other nutritional deficiencies that could limit the effects of B12 alone are covered in one arm of the trial.

We follow the Indian national nutritional anaemia prophylaxis programme guidelines for anaemia prevention [37]. All participants are separately administered weekly iron and folic acid tablets (100 mg iron and 500 μg folic acid) and the MMN tablet does not contain these micronutrients. All participants will be treated with a six month course of B12 at the end of the study.

### Sample size and power

We calculated the power of the study from observational data from Pune relating maternal B12 concentrations to cord blood B12 concentrations; a 1 SD higher maternal B12 concentration was associated with a 0.5 SD higher cord blood B12 concentration. Our earlier pilot intervention study (in men, non-pregnant women and children) showed that a dose of 2 μg of B12 led to a rise in B12 concentrations of approximately 1 SD over one year, with most of that increase achieved within 4 months [34]. Based on 180–200 pregnancies in the female participants, the trial will have 78–82% power to detect a change in newborn B12, with B12 supplementation compared with placebo, of 0.5 SD, at the 5% significance level.

Inclusion criteria:

- Adolescents (~16–18y) belonging to the original PMNS cohort

Exclusion criteria:

- Severe vitamin B12 deficiency (<100 pmol/L) or severe anaemia (haemoglobin < 7 g/dL) (due to ethical considerations in a placebo controlled trial)
- Severe physical and mental disability likely to interfere with marriage and reproduction
- Serious systemic illness (that would prohibit participation in any clinical trial e.g., malignancy, reproductive disorder leading to infertility, congenital or acquired cardiovascular disease with New York Heart Association Functional Classification III or IV)
- Treatment with drugs that interfere with 1-C metabolism (e.g., those interfering with folate metabolism such as phenytoin, valproic acid, carbamazepine, trimethoprim, methotrexate and those interfering with B12 absorption such as metformin, proton pump inhibitors)
- Treatment with B12 supplements for more than 30 days at the time of randomisation.

The participants will receive supplements for at least 3 years or until the first delivery (girls) and for the boys, until their wives become pregnant. The children born to these young men and women will be followed up at birth and also later for growth and measurements of diabetes risk markers.

### Consenting process

At the start of the study, detailed information and counselling was provided to the parents and children by investigators, co-investigators or trained social workers at home or in small groups. The children and parents were invited to a local village centre for detailed explanation of the project and the consent process, with transport provided. They had sufficient opportunity to ask questions for further clarification before agreeing to participate. For married participants, we also obtain consent from spouses for collecting cord blood. When obtaining consent from the participants, we have ensured that consent would cover use of the data to examine scientific questions outside the immediate remit of this study, with assurance of anonymity and confidentiality, including archiving of blood and DNA for relevant testing for at least 20 years.

### Pre-intervention screening

A team of investigators, biochemists, medical officer and medical social workers visited the six PMNS villages. Screening procedures included height, weight and blood pressure measurements, a clinical medical examination, and a random blood sample. Of the 696 who were available and approached, 690 underwent screening, 133 were excluded (117 due to severe B12 deficiency [<100 pmol/L], 2 due to severe anaemia [<7 g/dL] and 14 due to medical conditions) leaving 557 eligible participants. Those who were excluded due to severe B12 deficiency and anaemia were treated appropriately.

### Randomisation

The 557 eligible participants were randomised individually into three groups. Randomisation was achieved by a computer generated random sequence numbering system using STATA.

### Blinding

The participants and the study team are blinded to the vitamin/micronutrient supplementation. After randomisation, the list of names of participants in each allocation group was used by an independent staff member not involved in the day-to-day running of the study to package the bottles of capsules into boxes labelled only with the participants’ names (not with the allocation group). The bottles of supplements were labelled with the participants’ names and ID numbers along with information such as instructions for storage, lot number, expiry date, manufacturer details and a statement that these are for trial purposes and not for sale. The labels clearly highlighted that the contents were for the consumption of the individual named person only. The information on labels was based on guidance from the chair of the scientific advisory committee overseeing the trial. These measures ensured that all members of the research team in direct contact with participants or involved in outcome measurements are completely blinded to the allocation group. The capsule supplements for all groups are identical in appearance. The codified contents of the capsules are in the custody of the Director of Research, KEM Hospital Research Centre, and will be revealed only at the end of the study, except in the case of an emergency medical situation or severe adverse event requiring unblinding. We had a buffer stock of supplements to allow for lost bottles. Bottles of each individual participant were kept in a box labelled with similar information as on the bottles. The log of distribution of individual bottles and date of collection of the previously distributed bottles was maintained for each participant and the information duplicated on these boxes.

The milk powder intervention is not blinded. We initially intended to supply a placebo powder to the other groups, in order to blind the milk powder intervention. However, this was not possible, due to financial constraints. The field workers who distribute the supplements are unaware that it is only the MMN group that receives milk powder. All outcome assessments are done by staff who are blinded to the allocation groups.

### Intervention Product (IP) development

The dose of vitamin B12 (2 μg/day) was shown to be efficacious in our pilot trial [34]. The composition of the MMN tablet was guided by the WHO/UNICEF/UNU international multiple micronutrient preparation (UNIMMAP). It provides approximately 1 RDA of a range of vitamins and minerals, except iron and folic acid (which are administered separately to all groups). The final content was based on a pragmatic approach and a balance between Schedule V of the Drug and Cosmetic Act 1945 (amended; [38]), and the ICMR Recommended Dietary Allowance [39]. As the dose of vitamin B12 could not exceed 1 μg in a single tablet, to fit in with DCA regulations, we decided to administer two tablets of micronutrients supplements, each containing 1 μg of vitamin B12 (Table 1). A reputed local manufacturer was contracted to supply the B12, B12 plus MMN and placebo tablets in batches based on shelf life. Since the micronutrient supplement was considered a nutrient, and not a drug, Food and Drug Administration approval was not necessary.

**Table 1:** Contents of the multiple micronutrient supplement tablets, and comparison with the WHO/UNICEF/UNU international multiple micronutrient preparation (UNIMMAP), Schedule V of the Drug and Cosmetic Act (DCA) regulations, and the Indian Council of Medical Research (ICMR) RDA for Indians

	UNIMMAP (1999)	Schedule V, DCA (2003)	ICMR RDA (2010)		PRIYA
			Male	Female	
Vitamin A (μg)	800	480–750	600	600	300
Vitamin D (IU)	200	100–200	400		100
Vitamin E (mg)	10	3.35–6.7	7.5–10		5
Vitamin C (mg)	70	25–50	40	40	20
Vitamin K (μg)	–	–	55		–
Vitamin B1 (mg)	1.4	1–2	1.2–1.7	1.0–1.4	0.75
Vitamin B2 (mg)	1.4	1–3	1.4–2.1	1.1–1.7	0.9
Vitamin B3 (mg)	18	15–26	16–21	12–16	10
Vitamin B6 (mg)	1.9	0.5–1.5	2		0.5
Vitamin B12 (μg)	2.4	0.5–1	1.0	1.0	1.0
Zinc (mg)	15		12	10	6
Copper (mg)	2		2		1
Selenium (μg)	65		40		20
Iodine (μg)	150		150	150	75

*Dose in PRIYA 2 capsules/day to achieve 2* μg of Vitamin B12; B12 group tablet content is 1 μg of Vitamin B12 per capsule; placebo group tablet contains no micronutrients; iron and folic acid given separately to all groups

To comply with the Government programme of weekly supplementation with tablets of iron and folic acid (100 mg of elemental iron and 500 μg of folic acid), we took the responsibility of procuring and administering the recommended supplements to all participants.

We originally intended to supply 300 ml of milk to achieve the increase in protein intake. However due to safety and logistical issues, we explored alternative options (nutrient bars, cookies, powder). We eventually decided to administer 20 g of milk powder (containing 5 g milk protein) in sachets. This formulation has a good shelf life (2 years) and sachets can be distributed along with the micronutrients. The powder can be dissolved either in milk or water to produce a palatable drink.

All intervention products were kept in a separate secure room and the products were accessible only to specific staff members.

### Intervention

The intervention commenced in September 2012. All participants were visited individually and counselled again before being given the supplements. Participants receive supplements on a monthly cycle (with a buffer of 3 extra days’ supply). The protein supplements were started in May 2013.

### Compliance

Compliance is being monitored by retrieving the containers of the tablets and counting the number remaining in the container every month. Compliance is encouraged by follow-up telephone calls and a newsletter. We have used the monthly approach successfully in an earlier study which extended over a year, and at the end of which mean compliance remained 80% [34]. We therefore consider the monthly approach both workable within available resources and effective in our population.

### Monitoring of adverse events/morbidity

Vitamins in the dosages used are not expected to produce any adverse effects. Participants have been encouraged to contact the field social workers if they feel unwell or experience any adverse events, and in the event of hospitalisation for any reason. Otherwise adverse events and morbidity are monitored on a monthly basis using a standardised questionnaire. All relevant information about any illness is gathered, as well as details of any medication (self-administered or prescribed by a doctor). Serious adverse events (SAE; death, hospital admissions for more than 24 h, debilitating illness) are recorded and investigated by medical personnel overseen by the PI. Regular reports are submitted to the Ethics Committee and the Data Safety Monitoring Board (DSMB). On advice from the DSMB, hospital admission for delivery (>24 h) is also reported as an SAE, taking into account that it is also the outcome of interest.

### Out of area migrations

Participants who have migrated out of the original PMNS study area (approximately 60 sq. km.) but remain within the state of Maharashtra are included in the study. A total of 140 participants had migrated, mainly for education, marriage or work. The majority (105) were within Pune district and less than 70 km away. However, some had migrated outside the district and live at considerable distances from the study site; the farthest is ~700 km away. We have modified the distribution of the supplements to every two months for participants who have migrated within the district and participants who have migrated out of the district are given supplements for three months. We expect to be able to collect delivery data even for women who have married outside the study area, as almost all women return to their mother’s home for the delivery and for 1–3 months after the birth.

#### Study events during supplementation

##### Interim sample collection

We collected an interim sample (at least 6 months after start of supplementation) from April 2013. Its main purpose was to assess the efficacy of the supplementation in improving vitamin B12 status in the different groups. The data were analysed by an independent statistician at the MRC Lifecourse Epidemiology Unit, Southampton and results were sent directly to the Advisory and DSMB Committees, ensuring that investigators remained unaware of results for specific allocation groups. Based on the results, the committees reported to the investigators that the vitamin B12 intervention was efficacious and recommended continuation of the trial.

##### Marriage and pregnancy

After marriage, female participants and wives of male participants are monitored to detect pregnancy. This is being done by the field workers, who monitor menstrual dates. When participants report missing a period, a urine pregnancy test or an ultrasound scan is done to confirm pregnancy.

Once pregnancy is confirmed, the women (including the wives of male participants) are given and advised to take 1 tablet daily of 100 mg iron and 500 μg folic acid for 100 days to prevent anaemia, as per Indian Government guidelines. The study supplements are continued among the PMNS girls until their first delivery. Ultrasound scans are carried out at 28 weeks gestation, when blood pressure is also measured, and an OGTT performed (75 g anhydrous glucose). Samples for OMICs studies (blood, breast skin swab and stool) are also collected. Women diagnosed with gestational diabetes are appropriately treated. Local physicians manage the women as per routine standards of care, and we do not become involved unless asked to. We record all concomitant medication, including vitamin supplements.

Husbands of the female participants are offered anthropometric and blood tests at the time of the 28 weeks measurements (hemogram, blood glucose). Wives of the male participants are offered the same tests during pregnancy as the female participants in the study.

##### Delivery

We promote institutional delivery as per Indian Government recommendations. Obstetric care is provided by local doctors. We record relevant events and details of supplements/medication but decisions about obstetric care and management are made by the treating doctor, who is unaware of the allocation group. Maternal blood and samples for microbiota (vaginal swab, breast skin swab, and stool) are collected at delivery. Cord blood is collected for measurement of B12, folate and tHcy, and DNA and RNA samples stored. Placental weight, length and breadth are measured, and samples for molecular biology measurements and histopathology collected. Detailed neonatal anthropometry (including skinfold thickness) is carried out within 72 h. A newborn examination is performed by a paediatrician. Any neonatal medical problems are recorded in a questionnaire, and a newborn stool sample, as soon as possible after birth, is collected for microbiota. New-borns also undergo (since January 2015) a neonatal DXA for detailed body composition measurements.

##### Post-delivery

A paediatrician examines the baby at 28 days post-natally and information on breast feeding, any hospital admissions, and medication during the neonatal period are recorded.

##### Further plans

We originally intended to administer supplements for at least three years or until the first delivery. However, the number of marriages, and consequently pregnancies are lower than expected. Improving socioeconomic status and rising educational aspirations are some of the reasons for the later than predicted age of marriage in this rural area. We have therefore extended the study to cover intervention for at least five years or until delivery so that adequate numbers of deliveries take place to enable a proper assessment of the primary outcome. The Chairs of the Advisory and DSMB Committees have advised us that there is no cause for concern in continuing the trial as there are no significant adverse events related to the intervention. We have since secured permission from both ICMR and MRC, and ethical approval from our institutional ethics committee to continue the supplementation for a further two year period. Long-term follow-up, as in the PMNS itself, will start to document the growth and development, and cardiometabolic risk factors of children born in the study.

##### Laboratory measurements

**Fig. 4:**
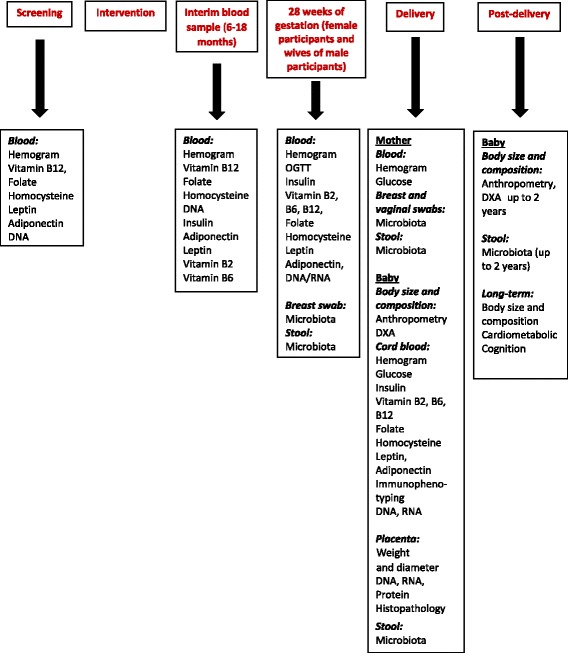
Sample collection at various time points in the trial

Figure 4 shows the samples collected at various time points. A part of the whole blood sample at all time points (EDTA vacutainer) is transferred to 2 ml micro centrifuge tubes for a haemogram. The remaining blood is centrifuged (2500 g × 15 min) within an hour of collection, and the separated plasma is stored at −70 °C until further analysis. Packed cells are stored for DNA; blood samples for RNA are collected into PAXgene tubes, extracted and stored at −70 °C. Cord blood is collected after delivery of the baby and clamping the cord. Up to 40 ml of free-flowing cord blood is collected in EDTA and heparin tubes by releasing the cord clamp. The heparin sample is processed for immunophenotyping within 6 h and the rest is processed as above. Samples for other OMICs studies (microbiota, immunophenotyping) and placental samples are also stored at −70 °C.

Biochemistry analyses are undertaken for various measurements:

- Hemogram: Beckman Coulter analyser (AC.T diffTM Analyzer, Miami, Florida, USA) on the day of collection.
- Plasma glucose and lipids: Automated biochemistry analyzer (Hitachi 902, Roche Diagnostics, Germany) using standard enzymatic kits.
- Insulin: ELISA kit (Mercodia AB, Uppsala, Sweden).
- Vitamin B2 and B6: standard kits (Recipe Chemicals + Instruments GmbH, Munchen, Germany) using HPLC (PerkinElmer 200 Series, PerkinElmer, Shelton, CT, USA) with fluorescence detector (B2 on whole blood; B6 on plasma).
- Vitamin B12: Microbiological assay using a colistin sulfate-resistant strain of L. Leichmanii.
- Folate: Microbiological assay using a chloramphenicol-resistant strain of L. Casei.
- Total Homocysteine, cysteine and glutathione: HPLC (PerkinElmer 200 Series, PerkinElmer, Shelton, CT, USA) using fluorescence detector.
- Leptin and adiponectin: ELISA kits (Alpco, Salem, NY, USA)

##### Data analysis

The primary short-term outcome (within the period of current funding) is cord blood vitamin B12. Other birth outcomes include birth weight and newborn body composition (by detailed anthropometry in all and by DXA where possible). The trial was set up to examine long-term outcomes, including growth, body composition, glucose and insulin parameters, cardiovascular disease risk markers, and cognitive function at intervals during childhood, and in adult life. The primary analysis will be by ‘*intention to treat*’ (ITT), only including newborns whose mothers received micronutrient supplementation for at least 3 months before conception. We will also undertake a per protocol analysis limiting the analysis to babies whose mothers had at least 50% compliance. We will undertake separate analyses for female and male participants.

We will adjust for vitamin B12 supplements prescribed outside the trial when analysing the primary outcome. We expect that randomisation will generate comparable groups but we will explore the effect of possible confounders and effect modifiers as appropriate for the other outcomes. We will adjust our analyses for maternal age, socio-economic status (before and after marriage), paternal BMI and height, gestational age at birth, and gender of the offspring. We will also examine for possible interaction between allocation group and the following pre-pregnancy factors: maternal BMI, height, age, pre-intervention B12 concentration; pregnancy factors: circulating folate, glucose and lipid concentrations; and fetal factors: newborn sex, and carry out stratified analyses when appropriate.

We will use analysis of variance to test for differences between group means. Appropriate transformations will be used to normalise data if required.

##### Data management

Field methods are regularly subjected to inter-observer and intra-observer variability exercises, and laboratory methods are subject to quality control using in-house methods (duplicates in assays, comparison with local standards) as well as national and international schemes (eg UK NEQAS schemes for insulin and BioRad EQAS schemes for haematology and biochemistry). Data entry is performed using Open Clinica, an open source software for clinical trial data management. Hard copies are checked by research staff, double entered onto computers and checked for quality. Hard data copies are stored in secure filing systems and standard systems are in place to ensure security of electronic formats. Any data transfer not carried out through protected servers is always encrypted. All data is anonymised before analysis and strict confidentiality is maintained. Analysis files (e.g., STATA or SPSS) are issued by the data manager only after approval from the PI, and a log is maintained.

##### Governance

We have gone to special lengths to clarify and discuss the ethical aspects of this study including the issues around conducting a placebo controlled trial in a community with substantial B12 deficiency as per international criteria. From a public health perspective, randomised studies are important because low B12 concentrations are common, and if B12 supplementation of mothers has clear benefits, this would indicate the need for national level intervention. On the other hand, from an individual point of view, it could be unethical not to treat people who, although apparently healthy, have B12 concentrations considered insufficient by international standards. The concept of a randomised controlled trial is unfamiliar to the villagers in our setting, many of whom are poorly educated and tend to trust medical professionals unquestioningly. We held a series of three meetings in Pune with ~30 lay people and scientists from different disciplines, focus group discussions with villagers, and meetings with a village Community Advisory Group made up of elders/leaders, teachers and participants in our research. We have also individually consulted experts in nutrition and public health research. The consensus was that the intervention study should proceed, because of the public health importance of getting an answer, combined with the individual-level considerations that a) the low B12 concentrations in this population are sub-clinical and evidence of its detrimental health effects is currently only observational and limited to risk markers; b) supplementation with B12 in the pilot trial produced no changes in haemoglobin concentration or other measurable individual health benefits; c) participants with B12 concentrations <100 pmol/l will be excluded and treated with B12 supplements, and d) the placebo group (all participants) will receive a 6 month course of B12 supplements at the end of the trial.

We also commissioned a study in the villages by an independent social scientist to explore the community’s understanding of research which suggested ways in which the study could be better explained to the participants. We have incorporated these findings in designing the information leaflets.

We follow ICMR and MRC (UK) guidelines for the ethical conduct of biomedical research. We have appointed a Scientific Advisory Committee (SAC) and a Data Safety Monitoring Board (DSMB) to oversee the trial, with appropriate membership. The Scientific Advisory Committee consists of clinicians, social scientists and medical administrators. The DSMB includes an epidemiologist, statistician, social scientist and nutritionist. Both are chaired by independent scientists with high level leadership experience in India. The SAC provides advice on the scientific aspects and conduct of the trial, and monitors its progress. The DSMB reviews data collection and quality, adherence to study protocol, safety records and adverse events and advises on further course of action including trial continuation. Reports are submitted every six months to both committees and a face-to-face meeting takes place annually. The trial has been registered with the CTRI (2012/12/003212) and ISRCTN (32921044).

##### Dissemination

Our main target audience will be the scientific community working in related fields. We will disseminate our findings through peer reviewed publications and presentations at national and international seminars/conferences. We will also extend our dissemination to scientists in other disciplines to whom our work will be relevant such as economists and agricultural scientists. We aim to publish our work in open access format. We will also engage with Indian and international policy makers. Members of our collaborative group are linked into national and international policy advisory groups such as the Indian Council of Medical Research, Department of Biotechnology (India), FIGO, WHO and UNICEF and we will ensure relevant findings are disseminated through these groups to influence policy.

As our participants are healthy volunteers, informing them about our findings has gained their support. Our research topics, including pregnancy, child health, and common adult disorders, are of general interest. We share information through community meetings, information leaflets and newsletters, and one-to-one feedback. We also disseminate our research to the wider public through a variety of mass media.

## Discussion

Our study tests a primordial prevention strategy through an intergenerational intervention started pre-conceptionally in both boys and girls using physiological doses of micronutrients to improve immediate pregnancy-related and long-term cardio metabolic outcomes. The results of our study will have potentially significant public health implications in an area where B12 deficiency is relatively common but folate deficiency is rare. The within cohort design and detailed information available will allow us to understand the lifecourse evolution of non-communicable diseases, and the randomised design will allow us to understand causal relationships.
